# Spectrum of *UGT1A1* Variations in Chinese Patients with Crigler-Najjar Syndrome Type II

**DOI:** 10.1371/journal.pone.0126263

**Published:** 2015-05-20

**Authors:** Lufeng Li, Guohong Deng, Yi Tang, Qing Mao

**Affiliations:** 1 Department of Infectious Diseases, Southwest Hospital, Third Military Medical University, Chongqing, China; 2 The Chongqing Key Laboratory for Research of Infectious Diseases, Chongqing, China; CSIR Institute of Genomics and Integrative Biology, INDIA

## Abstract

Crigler–Najjar Syndrome type II (CNS-II) is an autosomal recessive hereditary condition of unconjugated hyperbilirubinemia without hemolysis, with bilirubin levels ranging from 102.6 μmol/L to 342 μmol/L. CNS-II is caused by a deficiency of UDP-glucuronyl transferase (UGT), which is encoded by the UDP-glucuronyl transferase 1A1 gene (*UGT1A1*). In East Asian populations, the compound homozygous *UGT1A1* G71R and Y486D variants are frequently observed in cases with bilirubin levels exceeding 200 μmol/L. In this study, we investigated the spectrum of *UGT1A1* variations in Chinese CNS-II patients. We sequenced the enhancer, promoter, and coding regions of *UGT1A1* in 11 unrelated Chinese CNS-II patients and 80 healthy controls. Nine of these patients carried variations that are here reported for the first time in CNS-II patients, although they have been previously reported for other types of hereditary unconjugated hyperbilirubinemia. These individual variations have less influence on UGT activity than do the compound homozygous variation (combination of homozygous G71R variant and Y486D variant). Therefore, we propose that the spectrum of *UGT1A1* variations in CNS-II differs according to the bilirubin levels.

## Introduction

Inherited unconjugated hyperbilirubinemia is categorized as Gilbert Syndrome (GS; OMIM: 143500), Crigler–Najjar Syndrome type I (CNS-I; OMIM: 218800), or Crigler–Najjar Syndrome type II (CNS-II; OMIM: 606785), depending on the total bilirubin (TB) levels: the TB levels range from 17.1 μmol/L to 102.6 μmol/L, 102.6 μmol/L to 342 μmol/L, and 342 μmol/L to 769.5 μmol/L in GS, CNS-II, and CNS-I, respectively. CNS-I can be fatal due to kernicterus, which does not respond to phenobarbital treatment. However, the bilirubin level in CNS-II can be controlled by phenobarbital treatment to avoid the neurological damage caused by unconjugated bilirubin [1].

Liposoluble unconjugated bilirubin is glucuronidated to form hydrosoluble conjugated bilirubin by UDP-glucuronyl transferases (UGT) [2]. This enzyme is encoded by the UDP-glucuronyl transferase 1A1 gene (*UGT1A1*), which forms part of a complex gene locus (*UGT1A* gene complex). *UGT1A* encodes a number of UGTs by alternative splicing, each with various substrate preferences [3].

CNS-II is a rare autosomal recessive hereditary disease and is typically caused by rare homozygous or compound heterozygous mutations in *UGT1A1*. In some cases, CNS-II is caused by a combination of a heterozygous Crigler–Najjar-associated structural variant and a GS-associated variant in the promoter region of the gene (c.-40_-39insTA, rs3064744) [4]. To date, 45 *UGT1A1* variants have been reported in CNS-II patients [5]. However, most data originate from western countries. In Asia, the focus has predominantly been on clinical distinction between CNS-I and CNS-II and on patient response to phenobarbital and phototherapy.

We reviewed Crigler–Najjar case reports in NCBI PubMed, EMBASE, HGMD (CM062020, CM062020, CM941960, CD941964, CM931125, CM961403, CM972924, CM983519, CM002648, CM002649, CM002415, CD002537, CD014669, CM022853, CM051658, CM051659, CM051661, CM051662, CM051665, CM051666, CS051705, CS051706, CM066253, CM062021, CM062019, CD062241, CM067485, CM067484, CM100072, CM100073, CD100074, CM098937), OMIM (191740) databases between January 1992 (the year in which *UGT1A1* was first identified) and November 2014 and found that the compound homozygous G71R variant and Y486D variant were frequently detected in East Asian CNS-II patients whose bilirubin levels were greater than 200 μmol/L (10/13) [6–12].

Therefore, here, we studied the spectrum of *UGT1A1* variations in such patients, and compared the variations found in patients with bilirubin levels under 200 μmol/L with those variations reported for patients with levels exceeding 200 μmol/L.

## Materials and Methods

### Ethics Statement

The study protocol was approved by the institutional review board of the Southwest Hospital, Third Military Medical University (Chongqing, China). Written informed consent was obtained from all adult subjects or guardians of enrolled minors in the study.

### Patients and Controls

Patients were diagnosed with CNS-II using the following criteria: 1) a medical history of persistent unconjugated hyperbilirubinemia with TB > 102.6 μmol/L, comprising predominantly unconjugated bilirubin; 2) normal alanine transaminase and aspartate aminotransferase levels, to exclude hepatitis-related inflammation; 3) hemoglobin levels > 100 g/L (women) or 110 g/L (men) and reticulocyte counts < 1.5%, to exclude potential hemolysis; and 4) TB levels that were reduced with phenobarbital treatment, to distinguish CNS-II from CNS-I patients. Patients who had been on medication during the previous 3 months or who presented with comorbid diseases were excluded to avoid the influence of drugs.

Eleven unrelated patients with CNS-II were enrolled between April 2003 and May 2012 in the Department of Infectious Diseases, Southwest Hospital, Third Military Medical University (Chongqing, China). The bilirubin levels in nine of these patients were under 200 μmol/L (0.818). Aside from severe and persistent jaundice, the physical examination findings were remarkably negative; liver and spleen were not palpable, and no definite neurological abnormalities were described in any of the cases. The patients, who were all Han Chinese, did not appear to be ill, and all were asymptomatic. Clinical data of the patients are listed in Table 1.

**Table 1:** Clinical data and familial investigation of Crigler–Najjar syndrome type II cases in this study.

			Total Bilirubin / conjugated bilirubin (μmol/L)				
ID	Gender	Age of hyperbilirubinemia detected (Y)	Original prior to treatment	1 week after treatment	2 weeks after treatment	Healthy family members investigated	Comment
1	F	birth	286.4/12.8	200.4/11.8	120.4/5.8	1.1/1.2/1.6	
2	M	44	121/12.3	36/8.3	normal	2.1/2.2/2.5	
3	M	13	111/7.8	49/6.8		3.1	3.2 with GS
4	F	10	119.3/6.7	50.2/7.7	normal	4.1/4.2	
5	F	birth	515.1/13.6	390.9/16.8	150.3/12.8	5.1/5.2/5.3/5.4.1/5.4.2	5 underwent phototherapy at birth
6	M	16	150/12.4	83.1/7.9	normal	6.1/6.2	
7	M	10	104.3/9.2	74.1/13.5	normal	7.2/7.3	7.1 dead (cerebral hemorrhage) 7.4 with GS
8	M	13	120.1/7.4	77.1/15.1	normal	8.2/8.3/8.4	8.1 dead (lung disease)
9	M	29	131.1/9.9	62.0/16.1	normal	9.1/9.2	
10	M	15	171.5/13.6	89/12.6	normal	10.1/10.4	10.2 with GS 10.1 and 10.2 are first cousins
11	M	13	106.4/7.8	69.3/16.1	normal	11.1/11.2	

The numbers. 1,. 2,. 3,. 4,. 5, or. 6 following the patient number indicate father, mother, brother, sister, son, and daughter of the patient, respectively. ID = patient number; F = female; M = male; Y = years; GS = Gilbert syndrome

Eighty healthy controls were recruited from coworkers in the Department of Infectious Diseases, Southwest Hospital, Third Military Medical University (Chongqing, China). The healthy controls included 44 men and 36 women, with an average age ± standard deviation of 36.7 ± 11.4 y. All were Han Chinese. They underwent regular physical examination, including medical examination, blood biochemical examination, routine chest X-ray radiography, and abdominal ultrasonography examination at least every 2 years. Liver function tests, including those for TB and conjugated bilirubin, were performed, and the results were found to be normal during the time that they participated in the study.

### Nucleotide Sequencing of Coding and Functional Elements of *UGT1A1*

DNA was extracted from the peripheral blood of all subjects using the Wizard Genomic DNA Purification kit (Promega, Madison, WI, USA). PCR primers were designed for *UGT1A1* (GenBank accession number: NT_005120.16) using Primer 3.0 software (Whitehead Institute, Cambridge, MA, USA) and are detailed in the (S1 Table). PCRs were performed using a DNA Thermal Cycler (Bio-Rad, Hercules, CA, USA) under the following conditions: initial denaturation for 2 min at 95°C, 30 cycles each consisting of denaturation for 30 s at 94°C, annealing for 30 s at 58°C, and extension for 30 s or 40 s at 72°C, depending on the target size, and final extension for 7 min at 72°C. PCR mixtures contained Taq DNA polymerase (Promega, Madison, WI, USA) and Milli-Q water (Millipore, Billerica, MA, USA). PCR products were sequenced, using an ABI 3700 sequencer, by Beijing Genomics Institution (Beijing, China).

### Genotyping and Statistical Analyses

SNPs were identified using the DNASTAR software (DNASTAR Inc., USA) and inspected by at least two independent investigators. The SNP positions and individual genotypes were confirmed by re-amplifying and re-sequencing the SNP site from the same or opposite strand using the corresponding amplification primers. Statistical analysis was performed using SPSS PASW Statistics (version 18.0).

## Results

In this study, nine variants were detected (Table 2). The frameshift variant c.1253delT and nonsense variant c.715C>T are predicted to cause truncation of UGT (M418fsX423 and Q239X, respectively). The Y486D variant was detected only in CNS-II patients. The allele frequencies of the G71R variant and the P364L variant in CNS-II patients were greater than those of the controls (0.364 and 0.169, P = 0.042; 0.136 and 0.006; P = 0.006). The allele frequencies of the c.-3345delC variant, c.-3279T>G variant, and c.-40_-39insTA variant were not significantly different between the cases and controls (0.091 and 0.019, P = 0.111; 0.364 and 0.425, P = 0.584; 0.273 and 0.144, P = 0.128). The P229Q variant was only detected in heterozygous form in two controls.

**Table 2:** Variants and their allele frequencies in Crigler–Najjar syndrome type II cases and healthy controls.

			Allele frequency (the number of homozygotes/heterozygotes)			
Location	Nucleotide change	Predicted protein change	CNS-II (N = 11)	Control (N = 80)	P (x^2^ test)	dbSNP
Enhancer	c.-3345delC		0.091 (0/2)	0.019 (0/3)	0.111	rs34531096
Enhancer	c.-3279T>G		0.364 (3/2)	0.425 (13/42)	0.584	rs4124874
Promoter	c.-40_-39insTA		0.273 (2/2)	0.144 (1/21)	0.128	rs3064744
Exon 1	c.211G>A	p.G71R	0.364 (1/6)	0.169 (2/23)	0.042	rs4148323
Exon 1	c.686C>A	p.P229Q	0.000 (0/0)	0.013 (0/2))	1.000	rs35350960
Exon 1	c. 715C>T	p.Q239X	0.091 (0/2)	0.000 (0/0)	0.014	
Exon 4	c.1091C>T	p.P364L	0.136 (1/1)	0.006 (0/1)	0.006	rs34946978
Exon 4	c.1253delT	p.M418fsX423	0.045 (0/1)	0.000 (0/0)	0.121	
Exon 5a	c.1456T>G	p.Y486D	0.227 (2/1)	0.000 (0/0)	1.673E-05	rs34993780

Table 3 shows the *UGT1A1* genotypes detected in patients. Patients 1, 2, 3, 4, 5, 6, and 7 were found to carry homozygous or compound heterozygous variations, and the variations included at least one variant that causes a UGT truncation or one variant that is located in the shared exon of the *UGT1A* gene complex. These compound variations were not detected in the controls. Patient 1 was homozygous for both the G71R variant and the Y486D variant. Patient 2 was heterozygous for the G71R variant and homozygous for the Y486D variant. Patient 3 was homozygous for the P364L variant and heterozygous for the GS-associated promoter variant (c.-40_-39insTA). Patient 4 was heterozygous for both the Y486D variant and the M418fsX423 variant. Patients 5 and 6 were heterozygous for the Q239X variant and homozygous for the GS-associated promoter variant (c.-40_-39insTA). Patient 7 was heterozygous for both the P364L variant and the G71R variant. Patients 8 to 11 carried only a G71R structural variant. Patient 8 was also heterozygous for the GS-associated promoter variant (c.-40_-39insTA) and the c.-3279T>G variant in the enhancer, and this variation was also found in one control (Table 4). Additionally, a heterozygous c.-3345delC variant was found in the enhancer in patients 9 and 10, and this compound heterozygous genotype was not found in the controls. Patient 11 carried only the heterozygous G71R variant, and this variation was detected in seven controls in this study.

**Table 3:** *UGT1A1* genotypes of Crigler–Najjar syndrome type II cases.

Subject	Gender	Age (year)	TB/CB (μmol/L)	c.-40_-39insTA	Coding region	Enhancer
1	F	30	286.4/12.8	-/-	p.G71R+/+;p.Y486D+/+	Wild
2	M	49	121/12.3	-/-	p.G71R+/-;p.Y486D+/+	Wild
3	M	16	111/7.8	+/-	p.P364L+/+	c.-3279T>G+/+
4	F	15	119.3/6.7	-/-	p.M418fsX423+/-;p.Y486D+/-	Wild
5	F	12	515.1/13.6	+/+	p.Q239X+/-	c.-3279T>G+/+
6	M	21	150/12.4	+/+	p.Q239X+/-	c.-3279T>G+/+
7	M	16	104.3/9.2	-/-	p.G71R+/-;p.P364L+/-	c.-3279T>G+/-
8	M	49	120.1/7.4	+/-	p.G71R+/-	c.-3279T>G+/-
9	M	33	131.1/9.9	-/-	p.G71R+/-	c.-3345delC+/-
10	M	22	171.5/13.6	-/-	p.G71R+/-	c.-3345delC+/-
11	M	20	106.4/7.8	-/-	p.G71R+/-	Wild

F = female; M = male; Y = years

**Table 4:** *UG1A1* genotypes of 80 healthy controls.

c.-40_-39insTA	Coding regions	Enhancer	Frequency (N)
-/-	G71R+/-	c.-3279T>G+/-	0.188 (15)
-/-	Wild	Wild	0.163 (13)
-/-	Wild	c.-3279T>G+/-	0.163 (13)
+/-	Wild	c.-3279T>G+/-	0.150 (12)
+/-	Wild	c.-3279T>G+/+	0.075 (6)
-/-	G71R+/-	wild	0.088 (7)
-/-	Wild	c.-3279T>G+/+	0.050 (4)
-/-	Wild	c.-3345delC+/-	0.038 (3)
+/-	G71R+/-	c.-3279T>G+/-	0.013 (1)
+/-	P229Q+/-	c.-3279T>G+/-	0.013 (1)
+/-	P229Q+/-	c.-3279T>G+/+	0.013 (1)
+/+	Wild	c.-3279T>G+/+	0.013 (1)
-/-	G71R+/+	wild	0.025 (2)
-/-	p364l+/-	c.-3263T>G+/+	0.013 (1)

## Discussion

In this study, we investigated the spectrum of *UGT1A1* variations in 11 Chinese CNS-II patients and nine variants were identified. Nine of the 11 cases carried variations that are reported here for the first time in CNS-II patients (patients 3 to 11). The two variants predicted to cause truncation of UGT (M418fsX423 variant and Q239X variant) were also previously detected in CNS-I patients [13, 14]. The Y486D variant has been shown to lower the bilirubin UGT activity by more than 90% [15] and was detected only in CNS-II patients. Other variants of which the allele frequencies were greater than those of the controls (G71R variant and P364L variant) have also been shown to lower bilirubin UGT activities [8, 16], as does the c.-40_-39insTA variant [17]. All patients carried single or multiple copies of the above-mentioned variants (Table 3). A heterozygous P364L variant was previously reported in GS patients [8, 18]; however, the homozygous P364L variant was reported in this study for the first time. Besides an infant patient diagnosed with CNS-II, but whose bilirubin levels recovered to normal without treatment at four months of age [19], no previously reported CNS-II patient carried only a heterozygous G71R variant as seen in patient 11 in this study.

We reviewed Crigler–Najjar case reports in NCBI PubMed, EMBASE, HGMD and OMIM and found that the spectra are marked variable in different populations. The compound homozygous G71R variant and Y486D variant were detected in 14 of 27 East Asian CNS-II patients [6–12, 20–26], while only in one of 58 Caucasian CNS-II patients and in one of 14 other racial CNS-II patients from India, Pakistani and so on (East Asian patients listed in Table 5; Caucasian and other racial patients listed in (S2 Table: UGT1A1 genotype of Caucasian and other racial previous reported CNS-II cases).

**Table 5:** *UGT1A1* genotypes of 27 East Asian Crigler–Najjar syndrome type II cases, as reported in NCBI PubMed, EMBASE (using the search-term “Crigler–Najjar” between January 1992, when *UGT1A1* was firstly identified, and November 2014), HGMD (CM062020, CM062020, CM941960, CD941964, CM931125, CM961403, CM972924, CM983519, CM002648, CM002649, CM002415, CD002537, CD014669, CM022853, CM051658, CM051659, CM051661, CM051662, CM051665, CM051666, CS051705, CS051706, CM066253, CM062021, CM062019, CD062241, CM067485, CM067484, CM100072, CM100073, CD100074, CM098937), and OMIM databases (191740).

Subject	Ethnicity	Gender	age (year)	TB (μmol/L)	Variation	Reference
S1	Japanese	F	58	332	p.G71R+/+;p.Y486D+/+	[6]
S2	Japanese	F	60	154	p.G71R+/+;p.Y486D+/+	[6]
S3	Japanese	F	74	282	p.G71R+/+;p.Y486D+/+	[6]
S4	Japanese	F	53	238	p.G71R+/+;p.Y486D+/+	[6]
S5	Japanese	M	74	258	p.G71R+/+;p.Y486D+/+	[6]
S6	Japanese	M	5	251	p.G71R+/+;p.Y486D+/+	[7]
S7	Japanese		62.8	236.8	p.G71R+/+;p.Y486D+/+	[8]
S8	Japanese		62.8	236.8	p.G71R+/+;p.Y486D+/+	[8]
S9	Japanese		62.8	236.8	p.G71R+/+;p.Y486D+/+	[8]
S10	Korean	M	NA	164.16	p.G71R+/+;p.Y486D+/+	[9]
S11	Korean	F	14	242.82	p.G71R+/+;p.Y486D+/+	[9]
S12	Korean	F	3	155.61	p.G71R+/+;p.Y486D+/+	[9]
S13	Japanese	F	34	307.8	p.G71R+/+;p.Y486D+/+	[10]
S14	East Asian	F	35	152.2	p.G71R+/+;p.Y486D+/+	[26]
S15	Taiwanese	F	28	173	p.G71R+/-;p.Y486D+/+	[12]
S16	Korean	M	1	167.2	p.G71R+/-;p.Y486D+/+	[9]
S17	Korean	F	5	184.68	p.G71R+/-;p.Y486D+/+	[9]
S18	Japanese	NA	62.8	139.4	p.Y486D+/+	[8]
S19	Chinese	M	22	157.8	p.Y486D+/+	[20]
S20	Japanese	M	37	136.8	p.Y486D+/+	[21]
S21	Chinese	M	8/12	107.3	p.G71R+/-;p.F170^-^+/-p.Y486D+/-	[2]
S22	Taiwanese	M	22	479	p.R209W+/-;p.D396IfsX15+/-	[11]
S23	Japanese	M	51	333	p.R209W+/+	[6]
S24	Taiwanese	F	21	217	p.M204V+/+	[12]
S25	Japanese	M	64	116	p.Q331X+/-	[22]
S26	Chinese	NA	NA	108.5	c.-40_-39insTA+/+;p.G71R+/-;p.P229Q+/-	[23]
S27	Japanese	M	8	167.5	c.-40_-39insTA+/+;p.H39D+/+	[24]

The *UGT1A1* enhancer was not sequenced in all cases; exclusions were patients S21, who carried a wild-type enhancer, and S27, who carried a homozygous c.-3279T>G variant in the enhancer. The *UGT1A1* promoter in patients S6, S10, S11, S12, S16, S19 was also not sequenced; patients S1, S2, and S3 are sisters; patients S7, S8, S9, and S18 (three males and one female) are subjects from the same study, and their detailed clinical data were not available to the authors: their mean age and bilirubin levels are 62.8 years and 236.8 μmol/L, respectively. The age of patient S10, the gender of patient S18, and the gender and age of patient S26 were not available. F = female; M = male; TB = total bilirubin; NA = not available

In these reported East Asian CNS-II cases, the TB levels of the CNS-II patients increased in the following order with respect to the *UGT1A1* variation detected: single variant homozygous Y486D variant, combination heterozygous G71R variant and homozygous Y486D variant, and compound homozygous G71R variant and Y486D variant (144.7 μmol/L, 175.0 μmol/L, and 232.0 μmol/L, respectively). Therefore, in East Asian populations, the Y486D variant is the main pathogenic cause of CNS-II in patients whose bilirubin levels exceed 200 μmol/L, and the GS-associated G71R variant[8, 27] is a synergistic factor for the unconjugated hyperbilirubinemia phenotype.

The previously reported spectrum of *UGT1A1* variations is distinct from the spectrum of *UGT1A1* variations reported here. Our study found many variants that have a less marked influence on UGT activity than does the Y486D variant. This variant lowers the bilirubin UGT activity by more than 90% [15], while the c.-3279T>G variant, c.-40_-39insTA variant, G71R variant, and P364L variant reduces bilirubin UGT activity to 62% [28], 18–33% [17], 40% [16], and 36.6% of normal [8] respectively. The distinction is evident from significant differences in bilirubin levels. The TB levels of the majority of cases (0.818) in this study (except for patients 1 and 5), were less than 200 μmol/L, and the mean TB levels of these nine cases were significantly lower than the mean TB levels of 14 previously reported cases carrying the compound homozygous G71R variant and Y486D variant (126.1 μmol/L and 232.0 μmol/L; P = 3.084E-05).

**Fig 1:**
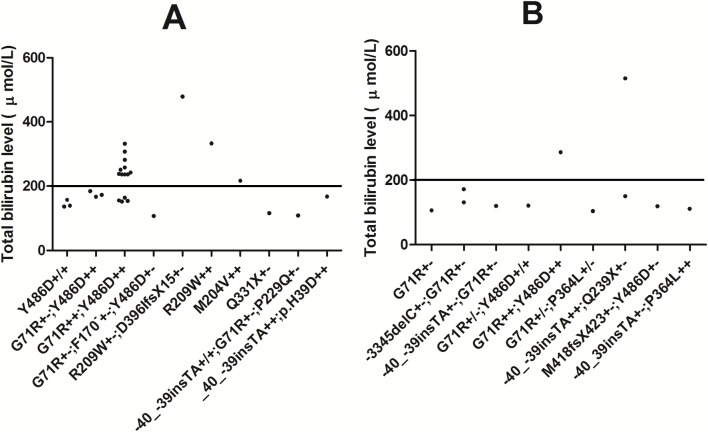
Different spectra of *UGT1A1* variations for CNS-II according to different bilirubin levels. A. Previously reported spectrum of *UGT1A1* variations for CNS-II. Bilirubin levels exceed 200 μmol/L in 13 of the 27 cases (0.481); B. Novel spectrum of *UGT1A1* variations for CNS-II in the present study. Bilirubin levels are under 200 μmol/L in 9 of the 11 cases (0.818).

However, the two spectra are comparable in terms of the relationship between *UGT1A1* variation and bilirubin levels (Fig 1). The common compound homozygous G71R variant and Y486D variant occur frequently in CNS-II patients who have bilirubin levels greater than 200 μmol/L, but rarely occur in patients with levels less than 200 μmol/L (10 vs 4 in the previously reported CNS-II spectrum; 1/0 in the spectrum reported here). Moreover, similar variations appear in patients with bilirubin levels both greater or less than 200 μmol/L (patient 1 and patients S1, S3 to S9, S11, S13; patient 2, and patients S15 to S17). For other patients in the two series (patients 3, 4, and 5 to 11, and patients S2, S10, S12, S14, S18 to S27), variations detected in all patients with bilirubin levels exceeding 200 μmol/L (patients S22 to S24) were consistent with the rule concluded by Kadakol et al. (homozygous or compound heterozygous mutations in *UGT1A1*) [4], while some patients with bilirubin levels under 200 μmol/L carry a single heterozygous variation (patient 11 and patient S24).

The c.-40_-39insTA variant and the G71R variant are associated with GS, and the heterozygous Y486D variant or P364L variant have also been observed in GS patients previously [8, 27] These *UGT1A1* variants were all identified in CNS-II patients in this study. However, the spectrum of variation in this study was also distinct from the spectrum previously noted for GS. In a Japanese study, the variants that cause a truncation of UGT were absent in GS patients, and variants that are located in the shared exon of the *UGT1A* gene complex were only observed in 14.1% of GS patients (9/64) [8]. In a Taiwanese study, variants causing premature truncation of UGT or located in the shared exon of the *UGT1A* gene complex were absent from GS patients [27]. In this study, seven patients carried homozygous or compound heterozygous variations, including at least one variant that results in a truncation of UGT or is located in a shared exon of the *UGT1A* gene complex (0.636). These variants can cause the absence or deficiency of the conserved carboxyl terminal of all UGTs, leading to a significant loss of activity in all of the UGT1A subfamily proteins.

*UGT1A1* encodes only bilirubin UGT [29]. The activity of bilirubin UGT was found to be only 10% of normal in CNS-II liver tissue [29]. However, the variations identified in CNS-II patients whose bilirubin levels are less than 200 μmol/L do not reduce the UGT activity to 10% of normal, as estimated by previous studies [8, 15–17, 28]. This indicates that the TB level is relevant to UGT activity. Bilirubin UGT activity may be variable in CNS-II patients whose bilirubin levels are less than 200 μmol/L.

CNS-II is an autosomal recessive hereditary disease, and patients 11 and S24 carried only single heterozygous variants in *UGT1A1*. Bilirubin levels are influenced by other factors besides genetic variation, such as alcohol, fasting, stress, and medication [29]. During brisk hemolysis, TB levels are also generally lower than 68.4 μmol/L [30] It may be that other factors could lead minor fluctuations in bilirubin levels under some conditions, but such effects would be limited and temporary. The age of onset of nine of the 11 patients was less than 16 years, and all patients had jaundice for at least 3 years prior to enrollment (Table 1). In addition, patients in whom the phenotype could have been confounded by factors such as alcohol, fasting, stress, and medication were excluded from the study. In these cases, an unknown variation in another gene may exist in combination with the variation in *UGT1A1*. It has been reported that CNS-II cases carrying simultaneously *UGT1A1* and other gene variations in recent years [31–33].

The study cautions clinicians that with CNS-II there is a potential risk of drug toxicity and that different bilirubin levels can indicate a corresponding potential disorder of drug metabolism. The c.-40_-39insTA variant, G71R variant, and Y486D variant can influence the metabolism of irinotecan [34–37]. The Y486D variant can also affect flutamide glucuronidation [38]. The P364L variant could give rise to adverse effects of various drugs, such as acetaminophen [39]. Patients with TB levels greater than 200 μmol/L frequently carry the compound homozygous G71R variant and the Y486D variant (Fig 1). In contrast, patients with TB levels ranging from 102.6 to 200 μmol/L carry a broader spectrum of *UGT1A1* variants.

## Supporting Information

S1 Table Primers for UGT1A1 amplification and sequencing (including enhancer, promoter and all coding regions: exon 1, exon 2, exon 3, exon 4, exon 5b and exon 5a). (DOCX)

S2 Table UGT1A1 genotypes of Caucasian and other racial Crigler–Najjar syndrome type II cases apart from East Asian, as reported in NCBI PubMed, EMBASE (using the search-term “Crigler–Najjar” between January 1992, when UGT1A1 was firstly identified, and November 2014), HGMD (CM062020, CM062020, CM941960, CD941964, CM931125, CM961403, CM972924, CM983519, CM002648, CM002649, CM002415, CD002537, CD014669, CM022853, CM051658, CM051659, CM051661, CM051662, CM051665, CM051666, CS051705, CS051706, CM066253, CM062021, CM062019, CD062241, CM067485, CM067484, CM100072, CM100073, CD100074, CM098937), and OMIM databases (191740). (DOCX)
