# Supplementary material for: Spectrum of UGT1A1 Variations in Chinese Patients with Crigler-Najjar Syndrome Type II
Source: PLoS One. 2015 May 20;10(5):e0126263. doi: 10.1371/journal.pone.0126263 (PMC4439166; doi:10.1371/journal.pone.0126263)
Supplement: S1 Table — (DOCX) [file pone.0126263.s001.docx]

**S1 Table. Primers for *UGT1A1* amplification and sequencing**

| **Region** | **Direction of Primers** | **Sequence (5' to 3')** | **Target Size**  **(base pair)** |
| --- | --- | --- | --- |
| **Enhancer** | **forward** | **TGCTATATAATGACGATGAATTTTGG** | **600** |
|  | **reverse** | **TTGCTCTCAAAACTCTGGGATA** |  |
| **Promoter and Exon 1** | **forward** | **ACTCCCTGCTACCTTTGTGGA** | **1167** |
|  | **reverse** | **TGCCAAAGACAGACTCAAACC** |  |
| **Exon 2** | **forward** | **AAACACGCATGCCTTTAATCA** | **500** |
|  | **reverse** | **CAGGGAAAAGCCAAATCTAAGG** |  |
| **Exon 3** | **forward** | **AAGTTGCCAGTCCTCAGAAGC** | **546** |
|  | **reverse** | **TGCAGAAGAAAATGTGGGTTG** |  |
| **Exon 4** | **forward** | **GTGGGGTATCTCAACCCACAT** | **632** |
|  | **reverse** | **GAGGCAGAACTGCTTGAACG** |  |
| **Exon 5a** | **forward** | **GCAGCCATGAGCATAAAGAGA** | **1299** |
|  | **reverse** | **TTCTGGGCGATGTGATTACTG** |  |
| **Exon 5b** | **forward** | **ATGTGGCTGTGCTCATGTGT** | **546** |
|  | **reverse** | **CATGATGCTCAGGTTATGTGG** |  |
