# Supplementary material for: Spectrum of UGT1A1 Variations in Chinese Patients with Crigler-Najjar Syndrome Type II
Source: PLoS One. 2015 May 20;10(5):e0126263. doi: 10.1371/journal.pone.0126263 (PMC4439166; doi:10.1371/journal.pone.0126263)
Supplement: S2 Table — (DOCX) [file pone.0126263.s002.docx]

S2 Table. *UGT1A1* genotypes of Caucasian and other racial Crigler–Najjar syndrome type II cases apart from East Asian, as reported in NCBI PubMed, EMBASE (using the search-term “Crigler–Najjar” between January 1992, when *UGT1A1* was firstly identified, and November 2014), HGMD (CM062020, CM062020, CM941960, CD941964, CM931125, CM961403, CM972924, CM983519, CM002648, CM002649, CM002415, CD002537, CD014669, CM022853, CM051658, CM051659, CM051661, CM051662, CM051665, CM051666, CS051705, CS051706, CM066253, CM062021, CM062019, CD062241, CM067485, CM067484, CM100072, CM100073, CD100074, CM098937), and OMIM databases (191740)

| Ethnicity | Origin | Gender | Age(Year) | TB(µmol/L) | Coding regions | TATA box | Reference |
| --- | --- | --- | --- | --- | --- | --- | --- |
| Caucasian | Portugal | NA | NA | 108 | G308E+/- | TA6/TA7 | [1] |
|  | Italy | NA | 3/12 | 124.66 | W354R+/- | TA6/TA7 | [2] |
|  | Italy | NA | 3/12 | 136.8 | I294T+/- | TA7/TA8 | [3] |
|  | Netherlands | NA | 4 | 140 | L175Q+/-;A325LfsX40+/- | NA | [4] |
|  | Italy | NA | 3/12 | 171 | L443P+/- | TA6/TA7 | [2] |
|  | USA | F | 1.6 | 171 | K407RfsX4+/- | TA6/TA7 | [5] |
|  | USA | F | 1.6 | 171 | K407RfsX4+/- | TA6/TA7 | [5] |
|  | USA | F | NA | 203.5 | L175Q+/+ | TA7/TA7 | [5] |
|  | USA | M | 10 | 205 | L496X+/- | TA6/TA7 | [5] |
|  | Germany | M | 21 | 234 | G71R+/+;Y486D+/+ | TA6/TA6 | [6] |
|  | Italy | M | 5 | 255 | E241GfsX5+/-;V225G+/- | TA7/TA7 | [7] |
|  | NA | M | 6 | 275 | wild type | TA7/TA7 | [8] |
|  | Italy | NA | 3/12 | 277.2 | W461R+/- | TA6/TA6 | [2] |
|  | USA | F | NA | 282 | L175Q+/+ | TA7/TA7 | [5] |
|  | France | NA | 6/12 | 300 | W354R+/-;G377V+/- | TA6/TA6 | [9] |
|  | Italy | NA | 3/12 | 306.1 | G395V;A409SfsX422+/- | TA6/TA6 | [2] |
|  | Italy | M | 13 | 308.1 | E241GfsX5+/-;V225G+/- | TA7/TA7 | [10] |
|  | Italy | NA | 3/12 | 324.9 | N279Y+/- | TA6/TA7 | [2] |
|  | France | NA | 4/12 | 332 | Q331R+/-;A429SX13+/- | TA6/TA7 | [9] |
|  | Netherlands | M | 40 | 340 | R209W+/+ | NA | [11] |
|  | Scotland | M | 72 | 342 | Q331R+/+ | TA6/TA6 | [12] |
|  | USA | M | 25 | 356 | E463A+/- | TA7/TA7 | [13] |
|  | Netherlands | F | 32 | 400 | L15R+/-；S191F+/- | TA7/TA7 | [14] |
|  | Netherlands | M | 23 | 467 | R336W+/-;A498PfsX3+/- | TA6/TA7 | [14] |
|  | Netherlands | M | 24 | 650 | R336W+/-;A498PfsX3+/- | TA6/TA7 | [14] |
|  | Italy | NA | NA | NA | A478D+/+ | TA7/TA7 | [15] |
|  | Italy | NA | NA | NA | F170del+/- | TA6/TA6 | [15] |
|  | Italy | NA | NA | NA | G377V+/-;V225G+/- | TA7/TA7 | [15] |
|  | Netherlands | M | 29 | NA | G71R+/-;Y486D+/+;K407RfsX4+/- | TA6/TA6 | [14] |
|  | Italy | NA | NA | NA | I294_S297delY293LfsX68+/- | TA6/TA7 | [15] |
|  | Italy | NA | NA | NA | I294_S297delY293LfsX68+/-;V225G+/- | TA6/TA7 | [15] |
|  | Italy | NA | NA | NA | I294_S297delY293MfsX68+/-;W354R+/- | TA6/TA7 | [15] |
|  | Portugal | NA | NA | NA | ISV1-1G>A+/-;G308E+/- | TA6/TA6 | [9] |
|  | Italy | NA | NA | NA | IVS1-1G>A+/-;R336L+/- | TA7/TA7 | [15] |
|  | Italy | NA | NA | NA | IVS4+1G>T+/-;R336W+/- | TA6/TA7 | [15] |
|  | France | NA | NA | NA | K407RfsX4+/- | TA6/TA7 | [9] |
|  | France | NA | NA | NA | K407RfsX4+/-;V225G+/- | TA6/TA7 | [9] |
|  | Netherlands | M | 33 | NA | L15R+/-;K407RfsX4+/- | TA6/TA7 | [14] |
|  | Netherlands | NA | NA | NA | L15R+/+ | NA | [16] |
|  | Netherlands | NA | NA | NA | L15R+/+ | NA | [16] |
|  | Netherlands | F | 33 | NA | L15R+/+ | TA7/TA7 | [14] |
|  | Netherlands | M | 19 | NA | L15R+/+ | TA7/TA7 | [14] |
|  | Netherlands | M | 17 | NA | L443P+/+ | TA6/TA6 | [14] |
|  | Italy | NA | NA | NA | P34Q+/-;R403C | TA6/TA6 | [15] |
|  | Netherlands | F | NA | NA | P387H+/- | TA6/TA6 | [14] |
|  | Russian | F | 37 | NA | P392L+/-;IVS1+5G>T/- | TA6/TA7 | [17] |
|  | Italy | NA | NA | NA | Q239fsX256+/-;V225G+/- | TA7/TA7 | [15] |
|  | Italy | NA | NA | NA | Q239fsX256+/-;W354R+/- | TA6/TA7 | [15] |
|  | France | NA | NA | NA | Q331R+/-;A429SX13+/- | TA6/TA6 | [9] |
|  | Italy | NA | NA | NA | R209W+/- | TA7/TA7 | [15] |
|  | Netherlands | M | 50 | NA | R209W+/+ | TA6/TA6 | [14] |
|  | Netherlands | F | 47 | NA | R209W+/+ | TA6/TA6 | [14] |
|  | Italy | NA | NA | NA | R336W+/- | TA6/TA7 | [15] |
|  | Italy | NA | NA | NA | R336W+/-;G377V+/- | TA6/TA6 | [15] |
|  | Italy | NA | NA | NA | V225G+/- | TA6/TA7 | [15] |
|  | France | NA | NA | NA | W354R+/-;G377V+/- | TA6/TA6 | [9] |
|  | France | NA | NA | NA | W40R+/-;ISV4+1G>T+/- | TA6/TA7 | [9] |
|  | Italy | NA | NA | NA | Y192X+/-;G377V+/- | TA6/TA7 | [15] |
| others | Morocco | NA | 3.5 | 173 | N400D+/+ | TA8/TA8 | [9] |
|  | Pakistani | NA | NA | 240 | Y230C | TA6/TA6 | [18] |
|  | Morocco | F | 3.5 | 306 | N400D+/+ | TA8/TA8 | [19] |
|  | Tunisia | NA | 8/12 | 340 | H132GN133_K134del+/+ | TA7/TA7 | [9] |
|  | Turkish | M | 3/12 | 343.7 | G71R+/-;K402T+/-;Y486D+/- | NA | [20] |
|  | India | M | 6/12 | 403.6 | G362S+/+ | TA6/TA6 | [21] |
|  | Iran | F | 11 | 444.6 | E241GfsX5+/+ | TA6/TA6 | [22] |
|  | Iran | M | 11 | 444.6 | V160E+/+ | TA6/TA6 | [22] |
|  | India | F | 16 | 461.7 | P170L+/+ | NA | [23] |
|  | Thailand | M | 4/12 | 494.02 | D119GfsX3+/- | TA7/TA7 | [24] |
|  | Iran | M | 9/12 | 533.5 | E241GfsX5+/+ | TA6/TA6 | [22] |
|  | African-American | M | 3 | 559.2 | M310V;1431T+/- | TA7/TA7 | [25] |
|  | Tunisia | NA | NA | NA | M1V+/+ | TA6/TA6 | [9] |
|  | Tunisia | NA | NA | NA | G71R+/+;Y486D+/+ |  | [9] |
|  | Turkey | NA | NA | NA | Q185P+/+ | TA7/TA7 | [9] |
|  | Malaysian | NA | NA | NA | G493R+/+ | NA | [26] |

F = female; M = male; TB = total bilirubin；NA = not available

**References**

1. Costa E, Vieira E, Martins M, Saraiva J, Cancela E, et al. (2006) Analysis of the UDP-glucuronosyltransferase gene in Portuguese patients with a clinical diagnosis of Gilbert and Crigler-Najjar syndromes. Blood Cells Mol Dis 36: 91-97.

2. D'Apolito M, Marrone A, Servedio V, Vajro P, De Falco L, et al. (2007) Seven novel mutations of the UGT1A1 gene in patients with unconjugated hyperbilirubinemia. Haematologica 92: 133-134.

3. Ciotti M, Chen F, Rubaltelli FF, Owens IS (1998) Coding defect and a TATA box mutation at the bilirubin UDP-glucuronosyltransferase gene cause Crigler-Najjar type I disease. Biochim Biophys Acta 1407: 40-50.

4. Seppen J, Bosma PJ, Goldhoorn BG, Bakker C, Chowdhury JR, et al. (1994) Discrimination between Crigler-Najjar type I and II by expression of mutant bilirubin uridine diphosphate-glucuronosyltransferase. Journal of Clinical Investigation 94: 2385.

5. Kadakol A, Sappal BS, Ghosh SS, Lowenheim M, Chowdhury A, et al. (2001) Interaction of coding region mutations and the Gilbert-type promoter abnormality of the UGT1A1 gene causes moderate degrees of unconjugated hyperbilirubinaemia and may lead to neonatal kernicterus. J Med Genet 38: 244-249.

6. Kraemer D, Klinker H (2002) Crigler-Najjar syndrome type II in a caucasian patient resulting from two mutations in the bilirubin uridine 5'-diphosphate-glucuronosyltransferase (UGT1A1) gene. J Hepatol 36: 706-707.

7. Minucci A, Canu G, Tellone E, Giardina B, Zuppi C, et al. (2012) Phenotype heterogeneity of hyperbilirubinemia condition: the lesson by coinheritance of glucose-6-phosphate dehydrogenase deficiency and Crigler-Najjar syndrome type II in an Italian patient. Blood Cells Mol Dis 49: 118-119.

8. Pena L, Pico M, Rosatelli C, Meloni A, Del Rio E, et al. (2012) UGT1A1 genotype in a white boy with Crigler-Najjar syndrome type 2. J Pediatr Gastroenterol Nutr 55: e136-137.

9. Petit F, Gajdos V, Capel L, Parisot F, Myara A, et al. (2006) Crigler-Najjar type II syndrome may result from several types and combinations of mutations in the UGT1A1 gene. Clin Genet 69: 525-527.

10. Iolascon A, Meloni A, Coppola B, Rosatelli MC (2000) Crigler-Najjar syndrome type II resulting from three different mutations in the bilirubin uridine 5'-diphosphate-glucuronosyltransferase (UGT1A1) gene. J Med Genet 37: 712-713.

11. Bosma PJ, Goldhoorn B, Oude Elferink RP, Sinaasappel M, Oostra BA, et al. (1993) A mutation in bilirubin uridine 5'-diphosphate-glucuronosyltransferase isoform 1 causing Crigler-Najjar syndrome type II. Gastroenterology 105: 216-220.

12. Moghrabi N, Clarke DJ, Boxer M, Burchell B (1993) Identification of an A-to-G missense mutation in exon 2 of the UGT1 gene complex that causes Crigler-Najjar syndrome type 2. Genomics 18: 171-173.

13. Chalasani N, Chowdhury NR, Chowdhury JR, Boyer TD (1997) Kernicterus in an adult who is heterozygous for Crigler-Najjar syndrome and homozygous for Gilbert-type genetic defect. Gastroenterology 112: 2099-2103.

14. Sneitz N, Bakker CT, de Knegt RJ, Halley DJ, Finel M, et al. (2010) Crigler-Najjar syndrome in The Netherlands: identification of four novel UGT1A1 alleles, genotype-phenotype correlation, and functional analysis of 10 missense mutants. Hum Mutat 31: 52-59.

15. Servedio V, d'Apolito M, Maiorano N, Minuti B, Torricelli F, et al. (2005) Spectrum of UGT1A1 mutations in Crigler-Najjar (CN) syndrome patients: identification of twelve novel alleles and genotype-phenotype correlation. Hum Mutat 25: 325.

16. Seppen J, Steenken E, Lindhout D, Bosma PJ, Elferink RP (1996) A mutation which disrupts the hydrophobic core of the signal peptide of bilirubin UDP-glucuronosyltransferase, an endoplasmic reticulum membrane protein, causes Crigler-Najjar type II. FEBS Lett 390: 294-298.

17. Passuello V, Puhl AG, Wirth S, Steiner E, Skala C, et al. (2009) Pregnancy outcome in maternal Crigler-Najjar syndrome type II: a case report and systematic review of the literature. Fetal Diagn Ther 26: 121-126.

18. Khan S, Irfan M, Sher G, Zubaida B, Alvi MA, et al. (2013) UGT1A1 gene mutations in Pakistani children suffering from inherited nonhemolytic unconjugated hyperbilirubinemias. Ann Hum Genet 77: 482-487.

19. Labrune P, Myara A, Chalas J, Le Bihan B, Capel L, et al. (2002) Association of a homozygous (TA)8 promoter polymorphism and a N400D mutation of UGT1A1 in a child with Crigler-Najjar type II syndrome. Hum Mutat 20: 399-401.

20. Maruo Y, Ozgenc F, Mimura Y, Ota Y, Matsui K, et al. (2011) Compound heterozygote of a novel missense mutation (p.K402T) and a double missense mutation (p.[G71R;Y486D]) in type II Crigler-Najjar syndrome. J Pediatr Gastroenterol Nutr 52: 362-365.

21. Aggarwal V, Seth A, Sharma S, Aneja S, Sammarco P, et al. (2010) Persistent jaundice in an infant with homozygous beta thalassemia due to co-inherited Crigler-Najjar syndrome. Pediatr Blood Cancer 54: 627-628.

22. Mohammadi Asl J, Tabatabaiefar MA, Galehdari H, Riahi K, Masbi MH, et al. (2013) UGT1A1 gene mutation due to Crigler-Najjar syndrome in Iranian patients: identification of a novel mutation. Biomed Res Int 2013: 342371.

23. Nair KM, Lohse P, Nampoothiri S (2012) Crigler-Najjar syndrome type 2: Novel UGT1A1 mutation. Indian J Hum Genet 18: 233-234.

24. Nilyanimit P, Krasaelap A, Foonoi M, Chongsrisawat V, Poovorawan Y (2013) Role of a homozygous A(TA)(7)TAA promoter polymorphism and an exon 1 heterozygous frameshift mutation UGT1A1 in Crigler-Najjar syndrome type II in a Thai neonate. Genet Mol Res 12: 3391-3397.

25. Ciotti M, Werlin SL, Owens IS (1999) Delayed response to phenobarbital treatment of a Crigler-Najjar type II patient with partially inactivating missense mutations in the bilirubin UDP-glucuronosyltransferase gene. J Pediatr Gastroenterol Nutr 28: 210-213.

26. Yusoff S, Van Rostenberghe H, Yusoff NM, Talib NA, Ramli N, et al. (2006) Frequencies of A(TA)7TAA, G71R, and G493R mutations of the UGT1A1 gene in the Malaysian population. Biol Neonate 89: 171-176.
